# The Examination of Different Tests for the Evaluation of the Efficiency of the Eggbeater Kicks

**DOI:** 10.2478/hukin-2014-0049

**Published:** 2014-07-08

**Authors:** Igor Stirn, Jernej Strmecki, Vojko Strojnik

**Affiliations:** 1 University of Ljubljana, Faculty of sport.

**Keywords:** water polo, testing, eggbeater kick, squat jump

## Abstract

The eggbeater kick presents an important basic technical skill in water polo. The aim of this study was to examine some different tests in order to recommend the best ones for the evaluation of the eggbeater kick. Twenty eight young male water polo players performed one test (squat jump) on land and ten tests in water: tethered swimming with legs only, using alternating and simultaneous eggbeater kicks, jumps out of water from basic and vertical (arms vertically above the head) position, water start and swimming two meters and swimming horizontally with legs only five meters with a flying start. The differences between tests were checked by executing dependent t-tests, while Pearson‘s correlation coefficients were calculated to evaluate the correlation between different parameters. Results showed that when performing alternate eggbeater kicks greater average forces were produced by the water polo players when compared to consecutive simultaneous eggbeater kicks. However, a short time maximal acceleration of the body in the vertical and horizontal plane was greater when the single simultaneous kick was performed. It was determined that horizontal swimming using legs only and a squat jump were less useful for the evaluation of the eggbeater kick. Therefore, the recommendation was to measure the average force of successive alternating eggbeater kicks, the height of the jump out of the water from the basic position and the water start and swim over a distance of 2 meters.

## Introduction

The egg beater kick is one of the most important technical skills in water polo. It is used to maintain the position of the body with the head above the water and to raise the body higher out of the water, to start the movement of the body in any direction, to jump vertically out of the water and to resist the opposing player when he/she is pushing in the opposite direction (Bratusa et al., 2003). An efficient eggbeater kick is also the prerequisite for a good passing and throwing technique (Stirn and Strojnik, 2006; McCluskey, 2010).

The skill of performing an effective eggbeater kick is important for all the players; however, it is the most important for the goalkeepers followed by the center forwards and center defenders and then wings and drivers (Dopsaj and Matković, 1999; Falk et al., 2004; Dopsaj and Aleksandrovic, 2009; Stirn, 2010). The descriptions of the time spent in the vertical position during the game are different for males and females. According to some studies a male water polo player spends from 55% to 66,9% in various options of the vertical position (Dopsaj and Thanopoulos, 2006; Dopsaj and Aleksandrović, 2009; Pinnington et al., 1988). However, when the FINA Women’s Water Polo World Cup in Perth in 2002 was analysed the time spent in the vertical position was approximately 23–40%, (14.0 ± 11.6% “wrestling”, and 8.9 ± 7.1% holding position). In the same study significant differences in wrestling time (9.9 ± 9.3% vs 18.4 ± 11.1%, p = 0.001) were found between outside and center players (D’Auria and Gabbett, 2008).

The eggbeater kick consists of a complex combination of hip and knee flexion and extension, hip adduction and abduction as well as hip internal and external rotation (Sanders, 1999; Homma and Homma, 2005). The eggbeater kick can be performed with both legs simultaneously (i.e. “boost”); or alternatively (i.e. “hold”); in this case a cyclical action is performed by both legs which is similar but opposite in the phase. The player can perform a single eggbeater kick (i.e. when starting); it is basically a breaststroke kick which consists of simultaneous (with both legs) hip and knee extension and hip internal rotation. A standard eggbeater kick is cyclical and it is used when maintaining a vertical position with arms out of water (“hold”), while the jump out of the water consists of the combination of both – first some alternate kicks are performed and the last kick is performed by both legs simultaneously to create maximal propulsion to raise the body as high as possible out of the water.

There is a lack of data in the literature regarding testing procedures of the eggbeater kick. Most often just the elevation of the upper body out of the water is used (Platanou, 2005; 2006; Stirn, 2010). However, based on the various manifestations of the eggbeater kick during a water polo game there is a need to evaluate the efficiency of this technical element under different conditions. Therefore, the aim of this study was to examine some different eggbeater kick tests and to examine the differences and correlations between different tests in order to determine whether they provide the same information or can contribute some additional insights into the skill.

## Material and Methods

Twenty-eight youth (14–16 years old) male water polo players participated in the study. They were invited to take part in testing as potential players of the U-16 national team. The study was approved by the Ethic Committee of Faculty of Sport, University of Ljubljana.

The players performed ten tests in the water while one test (squat jump) was performed on land. The tests were performed in random order.

**Figure 1:**
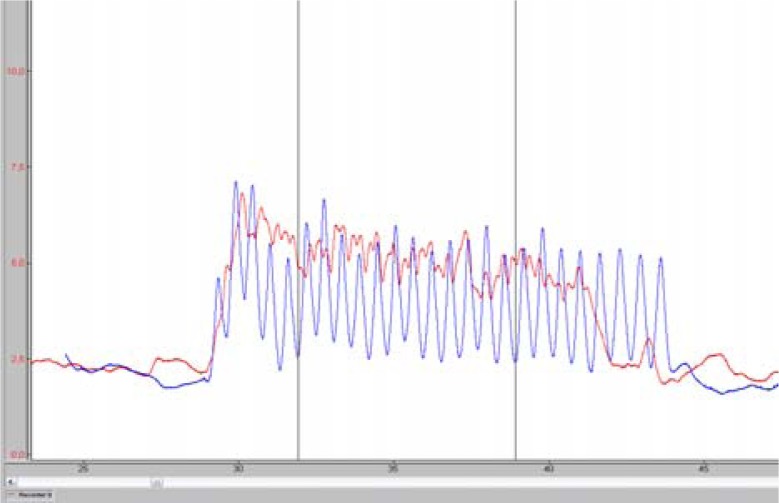
Force during tethered swimming: alternate (red) and simultaneous (blue) eggbeater kicks. Vertical lines mark the interval where the force was measured.

**Figure 2:**
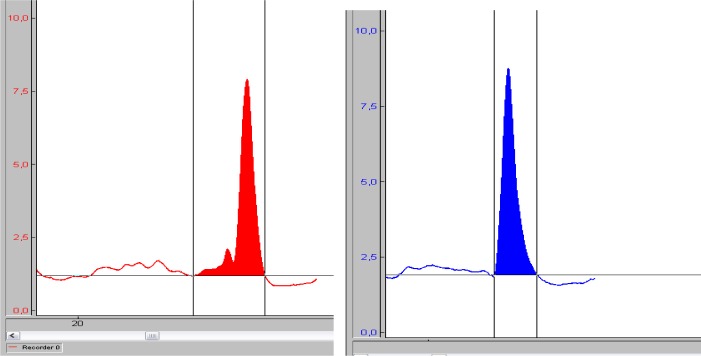
An example of the force recording of a single alternate eggbeater kick (left) and of a single simultaneous eggbeater kick (right). The filled area presents the force impulse.

Four of the tests consisted of tethered swimming with legs only. The player wore a waist belt and maintained the horizontal position in the water while holding the floating board in his arms in order to avoid arm movement (Picture 1). The inelastic cord was attached to the belt in one end and to the swimming pool edge at the other. In-between the pool edge and the cord the waterproof force sensor was connected to a custom made amplifier powered by batteries and it was mounted and connected to a notebook computer also operating on batteries. The player performed eggbeater kicks and strained the cord and the sensor. As a result of a strain the sensor voltage changed and was measured. The force sensor was calibrated using the weight of ten kilograms and this way the force was computed and expressed in Newton. The force of the pull on the cord was recorded and later analysed as shown in Figures 1 and 2.

In the position described above the player performed four tests:

1. Maximal pull for ten seconds with legs kicking alternatively – FTSa. The force recording is shown in Figure 1 – red line. First three seconds of force recording were not taken into account to eliminate the effect of the starting body inertia. The average value of the six-second interval marked with vertical lines was computed and subjected to further analysis.
2. Maximal pull for ten seconds with legs kicking simultaneously – FTSs. The force recording is shown in Figure 1 – blue line and was computed similarly as shown in the previous paragraph.
3. Maximal pull of the single alternate kick – FIa. The player was positioned as shown in Picture 1 and executed a single alternate eggbeater kick with a maximal effort. An example of the force impulse of a single alternate eggbeater kick is shown in Figure 2 (left). Maximal force and force impulse were measured. The test simulates one of the possible ways of performing the start of the movement of the body in horizontal position in the water.
4. Maximal pull of the single simultaneous kick - FIs. The player was positioned as shown in Picture 1 and executed a single simultaneous eggbeater kick with a maximal effort. An example of the force impulse of a single simultaneous eggbeater kick is shown in Figure 2 (right). Maximal force and force impulse were measured. The test simulates the other possible way of performing the start of the movement of the body in horizontal position in the water. Each player also performed six other tests:
5. Swimming with legs over 5 m with a flying start, using alternate eggbeater kicks – SW5a (Picture 2, left). The player swam over a 5 m distance with legs only (arms neutralised with the floating foam) executing alternating eggbeater kicks. The position of the foam was controlled in order to prevent excessive drag forces from the foam. The trial was recorded with the video camera with the frequency of 50 frames/second that was positioned 15 m away and above (from the balcony). This distance was chosen in order to minimise the effect of error when determining the moment of beginning and the end of swimming. The frames were counted from the moment when the player‘s head came in-line with the first mark to the moment when the head came inline with the second mark which was 5 m away. We calculated the swimming time by multiplying the number of frames by 0.02 s.
6. Swimming with legs only over 5 m with a flying start using a simultaneous eggbeater kick-SW5s (Picture 2, left). The procedure was similar to SW5a, yet the player performed simultaneous eggbeater kicks.
7. Swim start over a distance of 2 m – SS2m. The player maintained a horizontal position in the water similar to the situation at the start of each quarter in water polo game as shown in Picture 2 (right). At the sound signal of the whistle he executed a water start (a start of the movement of the body in a horizontal position) and swam over 2 meters. The trial was recorded with a video camera (50 Hz) positioned in line with the final 2 m mark. The number of frames from the moment when the head lost contact with the floating starting line at the start (not at the moment of sound signal) to the moment when the head was in line with the 2 m mark was counted and multiplied by 0.02 seconds to obtain the time.
8. Jump out of the water from the basic position – JBP. The player maintained the so called “basic goal-keeper position” under the measuring table and then jumped out of the water reaching the measuring table with one hand as shown in Picture 3 (left). Each trial was recorded with the video camera. The jumping height was read out from the recordings and the best result of the five trials was used for further analysis.
9. Jump out of the water from a vertical position performing alternating eggbeater kicks – JVPa. The player maintained a vertical position with the arms vertically above water so that the acromion reached the water surface. From this position the player raised the body as high as possible as shown in Picture 3 (right). Each trial was recorded with a video camera. The jumping height was read out from the recordings and the best result of the five trials was used for further analysis.
10. Jump out of the water from a vertical position performing simultaneous eggbeater kicks – JVPs. The protocol was the same as for the JVPa, yet, the player performed simultaneous eggbeater kicks.
11. Squat jump – SJ. Squat jumps were performed on the force plate (Kistler, Switzerland). The player stood on the force plate with the hip and knee joint flexed at 90° with hands on hips and jumped as high as possible. The highest of the three jumps was used for analysis. The jump height, take-off time, average acceleration and starting power (defined as the development of force after the first 100 ms) were analysed. Arm length can influence reaching height; therefore, we also calculated variables when arm length was deducted from the jump height and labelled it with R at the end of variable name (i.e. JVPa_R). The other possible variable that might have an influence on measured parameters was buoyancy. Therefore, we conducted a simple buoyancy test; the player held full breath, raised the arms above head in a vertical position and maintained it in the water. The length of the arms extended out of the water was measured and labelled bf. This length was then deducted from the jumping out of the water height.

Statistical analysis was conducted using the SPSS 14 software package. Initially descriptive analysis and standard normality tests (Kolmogorov-Smirnov) of the data were executed. The differences between similar tests when performing alternate or simultaneous eggbeater kicks were checked using dependent t-tests, while the correlations between different variables were calculated with the Pearson‘s correlation test.

## Results

Results of the descriptive analysis are presented in Table 2.

**Figure 3:**
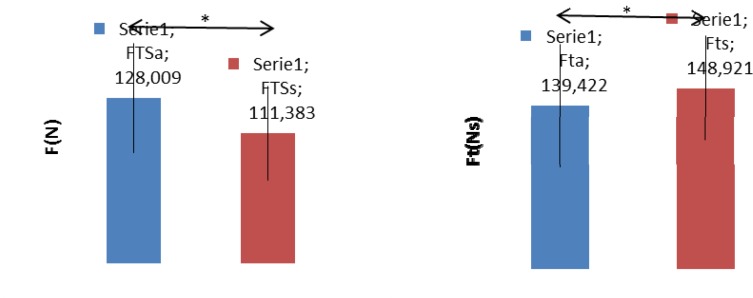
Left: Differences between the average forces measured during an alternate and simultaneous eggbeater kick. FTSa – average force during tethered swimming (alternate kicks), FTSs – average force during tethered swimming (simultaneous kicks). Right: Differences between the force impulse of a single alternate and single simultaneous eggbeater kick. Fta – force impulse of a single alternate eggbeater kick. Fts – force impulse of a single simultaneous eggbeater kick.

As shown in Figure 3 (left) the average force measured during tethered swimming with legs only while performing alternate eggbeater kicks (FTSa_avg) was larger than the same variable measured when simultaneous eggbeater kicks (FTSs_avg) were performed (128.01±25.85 N vs. 111.38± 22.02 N, p=0.001). When maximal forces are compared it can be observed that the forces measured during simultaneous kicks are larger than forces measured during alternating eggbeater kicks (p<0.001). This observation also stands for the comparison of maximal forces measured during the single alternate (FIa_max = 296.7 ± 80.4 N) and the single simultaneous (FIs_max = 360.5 ± 73.4 N) kick (p<0.001), while no differences (p=0.12) between the force impulses (Fta vs. Fts) of the single alternate and simultaneous kicks were observed (Figure 3, right)

The jumps out of the water with one hand are higher than the jumps out of the water from a vertical position with arms extended out of the water (p<0.001); however, there are no differences between latter whether they are performed with alternate or simultaneous eggbeater kicks (p=0.146).

The correlation matrix shows that average force measured during alternate kicks (FTSa_avg) is best correlated to the variables of tests JBP and JVPa. On the other hand force measured during simultaneous kicks was not correlated to jump out of water tests except for the low but significant correlation to the water horizontal start (SS2m). JVPs and horizontal swimming variables showed almost no correlations to any other parameters.

There was a medium size correlation (r=0.51 and r=0.53 respectively) found between the force impulse of a single eggbeater kick (Fta, Fts) and the jump out of water from a basic position. The water start test (SS2m) was correlated with the maximal force measured during a single alternate eggbeater kick (FIa_max) and with most JBP and JVPs parameters.

Parameters obtained during the squat jump in general were not found correlated to other tests, however, correlations of squat jump height and acceleration with simultaneous eggbeater kick variables corrected with the buoyancy factor were also found.

## Discussion

Our results show that when performing alternate eggbeater kicks greater average pushing forces were produced by the water polo players with respect to consecutive simultaneous eggbeater kicks. This suggests that the players should use this technique when they are “wrestling” with an opponent which is a common situation in modern water polo. This situation is especially typical for the duel between the center forward and 2-meter defender (Dopsaj and Matković, 1999; D’Auri and Gabbett, 2008). The goalkeepers are also using alternate eggbeater kicks when trying to maintain the high position of the body for a longer period of time. Sanders (1999) found that the force produced by the legs during the eggbeater kick is mainly dependent on the foot velocities during sculling. However, when a short time maximal acceleration of the body in vertical or horizontal plane is needed (i.e jump out of the water or the water horizontal start), the simultaneous kick should be used, as the maximal forces measured in our study during simultaneous kicks as well during a single eggbeater kick were the greatest. A typical technical situation of this type is the start of the movement of the body in the horizontal position in the water (water start).

Maximal forces as well as the force impulse of the simultaneous eggbeater kick were fairly correlated with the jump out of water from the basic position (Table 3). Indeed, when performing JBP, the players first perform fast consecutive alternate eggbeater kicks and then finally execute one single simultaneous kick when jumping out of the water (Sanders, 1999). When analysing this technique it becomes clear why JBP was correlated with both parameters. This observation suggests that the jump out of water from a basic position is a very important test.

Analysis of the single eggbeater kick was found demanding, as some excessive peak values could appear due to the body inertia effect at the beginning of the trial. Significant correlations with the jump out of the water from the basic position and the water start were found (both technical elements involve a single eggbeater kick). We propose these two tests for the evaluation of this ability.

Tests in which players swim with legs only in a horizontal position (executing alternating or simultaneous eggbeater kicks), showed poor or no significant correlations with other tests. Therefore, we determined that these tests were inappropriate for the evaluation of the efficiency of the eggbeater kick. This is an important observation, since this kind of a test or even breaststroke swimming is commonly used in practise to check for the “leg work” of the players and especially goalkeepers. This kind of a test actually measures (evaluates) the ability of gliding and not the ability of efficiently sculling the water using the eggbeater kick, which is very important for the swimming velocity of the breaststroker, but not for the water polo goalkeeper.

Similar observations stand for the obtained variables of the squat jump performed on land. It is well known that the squat jump is a powerful tool for lower limb power evaluations successfully used in many sports. No significant or poor correlations imply that these variables are not important for the water polo eggbeater kick. Similar conclusions were made by Platanou (2005) who found only a poor (r = 0.25) correlation between the land and water jump. Nevertheless, similar movements (hip and knee extension) are required in both jumps (in water and on land), yet, the abilities important for the squat jump are not relevant to the sculling eggbeater movement in the water. Jaric and Markovic (2009) showed that lower-limb muscles are predominantly designed to provide maximum dynamic output (assessed as power and momentum) in rapid movements like jumping against the load imposed by body mass and the inertia of their own body. In water body mass and inertia of the body are reduced due to the buoyancy. However, there were some correlations found with the parameters in water jump tests when the buoyancy factor was considered. On one hand, we could conclude that other factors, such as flexibility of the lower legs and ankles and eggbeater movement technique in general are more important for the efficiency of the eggbeater kick than leg functional strength. On the other hand, we can observe that the players were unskilled jumpers in general (average height 29.4 ± 3.8 cm) and that the differences among them in this skill were substantial. The reason for the low squat jump heights could also be in the above average body mass of our participants with respect to other athletes of the same age. Due to the buoyancy effect this fact does not prevent them from being successful in water polo, however, it will likely have an influence on squat jump results.

Endurance eggbeater tests were not the purpose of this research. Bampouras and Marrin (2010) reported that the repetitive 30 s crossbar jump test was not reliable. On the other hand, holding some additional weight in a vertical position with the arms extended above the head (elbows out of water) was found a useful test for the evaluation of endurance of the legs (Stirn, 2010).

Nevertheless, our study involved the players invited to the U-16 national team (expanded selection), negligible differences in the morphological characteristics, motoric abilities and technical skills were possible due to the small sample size of youth water polo players. Therefore, additional studies involving larger and more experienced (older) subjects should be performed in order to additionally confirm our findings.

## Conclusion

In conclusion, among the considered tests for the evaluation of the eggbeater kick in this study we recommend measuring the average force of successive alternating eggbeater kicks, height of the jump out of the water from the basic position, water start and swim over 2 m, while horizontal swimming tests and on land squat jumps were found less relevant. The data obtained with such measurements can be used in practise to evaluate players‘ abilities to perform the eggbeater kick and to monitor the training process.

## Figures and Tables

**Figure 4:**
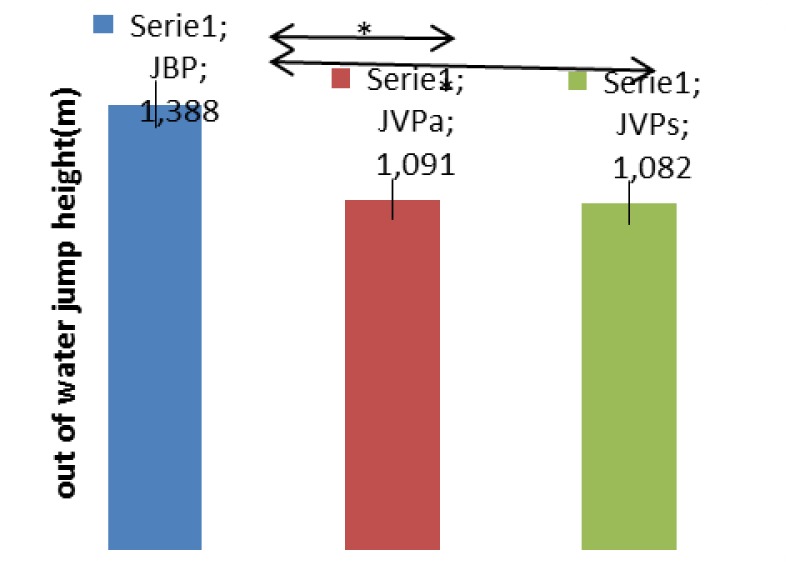
The differences between the jump out of water tests. JBP-jump from the basic position, JVPa-jump from the vertical position executing alternating kicks, JVPs-jump from the vertical position executing alternating kicks

**Picture 1:**
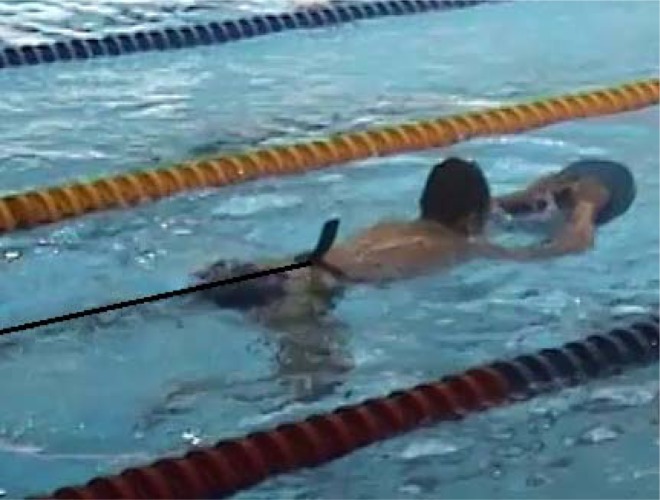
Tethered swimming with legs only, legs executing eggbeater kicks.

**Picture 2:**
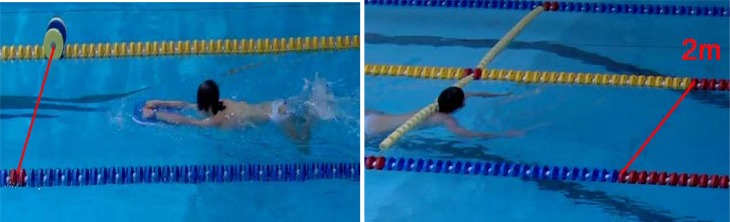
Swimming horizontally over the distance of 5 m using legs only with a flying start (left). Start of the movement of the body in the water and swim over the distance of 2 m (right).

**Picture 3:**
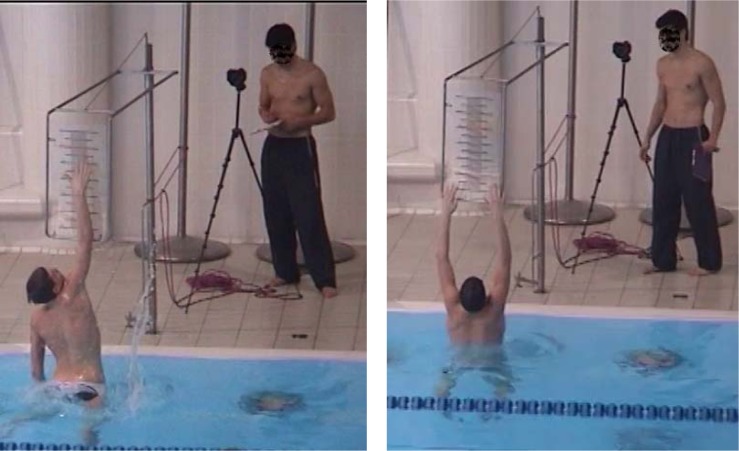
Jump out of the water from the basic position (left) and from the vertical position with arms extended out of the water.

**Table 1:** A list of tests used in the study

**Test**	**Abbreviation**
Maximal pull for 10 s with legs kicking alternatively	FTSa
Maximal pull for 10 s with legs kicking simultaneously	FTSs
Maximal pull of the single alternate kick	FIa
Maximal pull of the single simultaneous kick	FIs
Swimming with legs only over 5 m with a flying start using alternate eggbeater kicks	SW5a
Swimming with legs only over 5 m with a flying start using simultaneous eggbeater kicks	SW5s
Start and swim over the distance over 2 m	SS2m
Jump out of the water from the basic position	JBP
Jump out of the water from the vertical position performing alternating eggbeater kicks	JVPa
Jump out of the water from the vertical position performing simultaneous eggbeater kicks	JVPs
Squat jump	SJ

**Table 2:** Descriptive statistics

	Unit	Average	Min	Max	S.D.
Body height	[m]	1.800	1.690	1.960	0.067
Body mass	[kg]	75.575	54.900	100.700	12.601
FTSa_avg	[N]	128.009	88.818	179.770	25.853
FTSa_max	[N]	188.766	121.251	260.106	35.603
FTSs_avg	[N]	111.383	67.107	159.979	22.015
FTSs_max	[N]	243.446	160.726	309.450	37.054
FIa_max	[N]	296.728	168.207	502.065	80.435
Fta	[Ns]	139.422	86.033	214.728	33.647
FIs_max	[N]	360.524	227.935	567.880	73.431
Fts	[Ns]	148.921	100.489	237.065	28.379
JBP	[m]	1.388	1.290	1.520	0.072
JBP_R	[m]	0.572	0.437	0.676	0.065
JBP_bf	[m]	1.136	0.890	1.285	0.121
JVPa	[m]	1.091	1.010	1.210	0.062
JVPa _R	[m]	0.272	0.142	0.406	0.058
JVPa _bf	[m]	0.833	0.630	1.010	0.115
JVPs	[m]	1.082	0.950	1.255	0.069
JVPs _R	[m]	0.264	0.157	0.425	0.070
JVPs_bf	[m]	0.833	0.595	1.115	0.126
SW5a	[s]	6.245	5.410	7.950	0.643
SW5s	[s]	5.319	4.770	6.810	0.544
SS2m	[s]	1.389	1.100	1.800	0.173
SQJH	[m]	0.294	0.227	0.389	0.038
SQJT	[ms]	417.57	292.21	542.13	57.91
SQJA	[m/s^2^]	5.879	3.964	9.457	1.115
SQJSF	[N/kg]	1.286	0.300	2.900	0.676

FTSa_avg- average force during tethered swimming (alternate kicks), FTSa_max- maximal force during tethered swimming (alternate kicks), FTSs_avg- average force during tethered swimming (simultaneous kicks), FTSs_max- maximal force during tethered swimming (simultaneous kicks), FIa_max – maximal force during a single alternate kick, FIa_max – maximal force during a single alternate kick, FTa – force impulse of a single alternate kick, FIs_max – maximal force during a single simultaneous kick, FTs – force impulse of a single simultaneous kick, JBP – the height of the jump from the basic position, JBP_r – the relative height of the jump from the basic position, JBP_bp – the height of the jump from the basic position corrected by a buoyancy factor, JVPa – the height of the jump from the vertical position (alternate kicks), JVPa_r – the relative height of the jump from the vertical position (alternate kicks), JBPa_bp – the height of the jump from the vertical position corrected by a buoyancy factor (alternate kicks), JVPs – the height of the jump from the vertical position (simultaneous kicks), JVPs_r – the relative height of the jump from the vertical position (simultaneous kicks), JBPs_bp – the height of the jump from the vertical position corrected by a buoyancy factor (simultaneous kicks), SW5a – swimming five meters with legs only performing alternate kicks, SW5s – swimming five meters with legs only performing simultaneous kicks, SS2m - swimming two meters with water start, SQJH – squat jump height, SQJT – squat jump push off time, SQJA – squat jump acceleration, SQJSF- relative force at the start of the squat jump.

**Table 3:** Correlation between parameters of different eggbeater kick tests

	JBP	JBP_R	JBP_bf	JVPa	JVPa_R	JVPa_bf	JVPs	JVPs_R	JVPs_bf	SW5a	SW5s	SS2m
FTSa_avg	.611^**^	.554^**^	.540^**^	.457^*^	.399^*^	.437^*^				−.261		−.322
FTSa_max	.692^**^	.613^**^	.464^*^	.589^**^	.512^**^	.389				−.303		−.413^*^
FTSs_avg	.355	.338	.386				.253	.209	.272		−.329	−.392^*^
FTSs_max	.080	.212	.332				.155	.271	.325		−.146	−.153
FIa_max	.393^*^	.400^*^	.298	.176	.139	.156				−.381		−.482^*^
Fta	.512^**^	.441^*^	.323	.389^*^	.252	.209				−.230		−.473^*^
FIs_max	.320	.352	.226				−.072	−.110	−.056		−.347	−.145
Fts	.527^**^	.403^*^	.218				−.020	−.190	−.132		−.102	−.092
SW5a	−.280	−.411^*^	−.262	−.140	−.318	−.232						
SW5s	−.259	−.392^*^	−.367				−.244	−.333	−.275			
SS2m	−.545^**^	−.715^**^	−.649^**^	−.304	−.443^*^	−.489^*^	−.065	−.086	−.350			

See Table 2 for the abbreviations

**Table 4:** Correlation between the parameters of the squat jump and parameters of eggbeater tests

	**JBP**	**JBP_R**	**JBP_bf**	**JVPa**	**JVPa_R**	**JVPa_bf**	**JVPs**	**JVPs_R**	**JVPs_bf**	**SW5a**	**SW5s**	**SS2m**
**SQJH**	.271	.343	.495^*^	−.148	−.128	.283	.090	.221	.537^*^	−.069	−.334	−.254
**SQJT**	−.096	−.160	−.206	.081	.046	−.097	−.033	−.094	−.312	.241	.281	.045
**SQJA**	.223	.277	.336	−.062	−.059	.203	.103	.171	.448^*^	−.141	−.306	−.123
**SQJSF**	.046	.036	.057	−.172	−.236	−.102	−.001	.040	.193	−.106	−.296	−.011

See Table 2 for the abbreviations.
